# Nationwide Harmonization Effort for Semi-Quantitative Reporting of SARS-CoV-2 PCR Test Results in Belgium

**DOI:** 10.3390/v14061294

**Published:** 2022-06-14

**Authors:** Lize Cuypers, Jannes Bode, Kurt Beuselinck, Lies Laenen, Klaas Dewaele, Reile Janssen, Arnaud Capron, Yves Lafort, Henry Paridaens, Bertrand Bearzatto, Mathieu Cauchie, Aline Huwart, Jonathan Degosserie, Olivier Fagnart, Yarah Overmeire, Arlette Rouffiange, Ilse Vandecandelaere, Marine Deffontaine, Thomas Pilate, Nicolas Yin, Isabel Micalessi, Sandrine Roisin, Veronique Moons, Marijke Reynders, Sophia Steyaert, Coralie Henin, Elena Lazarova, Dagmar Obbels, François E. Dufrasne, Hendri Pirenne, Raf Schepers, Anaëlle Collin, Bruno Verhasselt, Laurent Gillet, Stijn Jonckheere, Philippe Van Lint, Bea Van den Poel, Yolien Van der Beken, Violeta Stojkovic, Maria-Grazia Garrino, Hannah Segers, Kevin Vos, Maaike Godefroid, Valerie Pede, Friedel Nollet, Vincent Claes, Inge Verschraegen, Pierre Bogaerts, Marjan Van Gysel, Judith Leurs, Veroniek Saegeman, Oriane Soetens, Merijn Vanhee, Gilberte Schiettekatte, Evelyne Huyghe, Steven Martens, Ann Lemmens, Heleen Nailis, Kim Laffineur, Deborah Steensels, Elke Vanlaere, Jérémie Gras, Gatien Roussel, Koenraad Gijbels, Michael Boudewijns, Catherine Sion, Wim Achtergael, Wim Maurissen, Luc Iliano, Marianne Chantrenne, Geert Vanheule, Reinoud Flies, Nicolas Hougardy, Mario Berth, Vanessa Verbeke, Robin Morent, Anne Vankeerberghen, Sébastien Bontems, Kaat Kehoe, Anneleen Schallier, Giang Ho, Kristof Bafort, Marijke Raymaekers, Yolande Pypen, Amelie Heinrichs, Wim Schuermans, Dominique Cuigniez, Salah Eddine Lali, Stefanie Drieghe, Dieter Ory, Marie Le Mercier, Kristel Van Laethem, Inge Thoelen, Sarah Vandamme, Iqbal Mansoor, Carl Vael, Maxime De Sloovere, Katrien Declerck, Elisabeth Dequeker, Stefanie Desmet, Piet Maes, Katrien Lagrou, Emmanuel André

**Affiliations:** 1 National Reference Centre for Respiratory Pathogens, Department of Laboratory Medicine, University Hospitals Leuven, 3000 Leuven, Belgium; jannes.bode@telenet.be (J.B.); kurt.beuselinck@uzleuven.be (K.B.); lies.laenen@uzleuven.be (L.L.); klaas.dewaele@uzleuven.be (K.D.); reile.janssen@uzleuven.be (R.J.); els.dequeker@uzleuven.be (E.D.); stefanie.desmet@uzleuven.be (S.D.); katrien.lagrou@uzleuven.be (K.L.); emmanuel.andre@uzleuven.be (E.A.); 2 Epidemiology of Infectious Diseases and Quality Service Unit, Scientific Directorate of Epidemiology and Public Health, Sciensano, 1000 Brussels, Belgium; arnaud.capron@sciensano.be (A.C.); yves.lafort@sciensano.be (Y.L.); 3 Clinical Laboratory, Centre Hospitalier Régional de la Citadelle, 4000 Liège, Belgium; henry.paridaens@chrcitadelle.be; 4 Federal Testing Platform COVID-19, Centre des Technologies Moléculaires Appliquées (CTMA), Institute of Experimental and Clinical Research (IREC), Cliniques Universitaires Saint-Luc and Université Catholique de Louvain (UCLouvain), 1200 Brussels, Belgium; bertrand.bearzatto@uclouvain.be; 5 Europe Hospitals, 1180 Brussels, Belgium; m.cauchie@europehospitals.be; 6 Clinique André Renard, 4040 Herstal, Belgium; aline.huwart@andrerenard.be; 7 Federal Testing Platform COVID-19, Department of Laboratory Medicine, CHU UCL Namur, 5530 Yvoir, Belgium; jonathan.degosserie@chuuclnamur.uclouvain.be; 8 Saint-Jean Hospital Laboratory, Cebiodi, 1000 Brussels, Belgium; o.fagnart@clstjean.be; 9 Microbiology, Labo Nuytinck, Anacura, 9940 Evergem, Belgium; yarah.overmeire@anacura.com; 10 Microbiology, Laboratory Luc Olivier, 5380 Fernelmont, Belgium; arlette.rouffiange@labolivier.be; 11 Microbiology, Medisch Labo Bruyland, 8500 Kortrijk, Belgium; ilse.vandecandelaere@bruyland.be; 12 Laboratory of Clinical Biology, Centre Hopsitalier de Mouscron, 7700 Mouscron, Belgium; m.deffontaine@chmouscron.be; 13 Clinical Laboratory, Laboratory Medicine, AZ Diest, 3290 Diest, Belgium; thomas.pilate@uzleuven.be; 14 Department of Microbiology, Laboratoire Hospitalier Universitaire de Bruxelles—Universitair Laboratorium Brussel (LHUB-ULB), Université de Bruxelles (ULB), 1000 Brussels, Belgium; nicolas.yin@lhub-ulb.be; 15 Clinical Reference Laboratory, Department of Clinical Sciences, Institute of Tropical Medicine, 2000 Antwerp, Belgium; imicalessi@itg.be; 16 Microbiology, Centre Hospitalier Universitaire de Tivoli, 7100 La Louvière, Belgium; sroisin@chu-tivoli.be; 17 Microbiology, LKO-LMC Medical Laboratory, 3800 Sint-Truiden, Belgium; veronique.moons@lkolmc.be; 18 Laboratory Medicine, AZ Sint-Jan Brugge-Oostende AV, 8000 Brugge, Belgium; marijke.reynders@azsintjan.be; 19 Clinical Laboratory, AZ Maria Middelares, 9000 Gent, Belgium; sophia.steyaert@azmmsj.be; 20 Federal Testing Platform COVID-19, Université Libre de Bruxelles, 1070 Brussels, Belgium; covidlab@ulb.be; 21 Centre Hospitalier Régional de la Haute Senne, Department of Clinical Biology, 7060 Soignies, Belgium; elena.lazarova@chrhautesenne.be; 22 Imelda, Clinical Laboratory, 2820 Bonheiden, Belgium; dagmar.obbels@imelda.be; 23 Federal Testing Platform COVID-19, University of Mons, 7000 Mons, Belgium; francois.dufrasne@sciensano.be; 24 Synlab Belgium, Synlab Laboratory Collard, 4020 Liège, Belgium; henri.pirenne@synlab.be; 25 Synlab Belgium, Synlab Laboratory Heppignies, 6220 Heppignies, Belgium; raf.schepers@synlab.be; 26 Vivalia, Site Marche-en-Famenne, 6900 Marche, Belgium; anaelle.collin@vivalia.be; 27 Federal Testing Platform COVID-19, Department of Laboratory Medicine, Ghent University and Ghent University Hospital, 9000 Gent, Belgium; bruno.verhasselt@uzgent.be; 28 Federal Testing Platform COVID-19, University of Liège, 4000 Liège, Belgium; l.gillet@uliege.be; 29 Jan Yperman Hospital, Laboratory of Clinical Biology, 8900 Ieper, Belgium; stijn.jonckheere@yperman.net; 30 GZA Hospitals, Clinical Laboratory, 2610 Wilrijk, Belgium; philippe.vanlint@gza.be; 31 Clinical Laboratory, General Hospital Jan Portaels, 1800 Vilvoorde, Belgium; bea.vandenpoel@azjanportaels.be; 32 Military Medicine Lab Capacity, Military Hospital Queen Astrid, 1120 Brussels, Belgium; yolien.vanderbeken@mil.be; 33 Centre Hospitalier Bois de l’Abbaye, Laboratory Service, 4100 Seraing, Belgium; v.stojkovic@chba.be; 34 Clinical Laboratory, Site Meuse, CHRSM, 5000 Namur, Belgium; mg.garrino@chrsm.be; 35 AZ Glorieux, Clinical Biology, 9600 Ronse, Belgium; hannah.segers@azglorieux.be; 36 RZ Heilig Hart Tienen, Clinical Biology, 3300 Tienen, Belgium; kevin.vos@rztienen.be; 37 Labo Maenhout, 8790 Waregem, Belgium; maaikegodefroid@labomaenhout.be; 38 AZ Sint-Elisabeth Zottegem, Laboratory of Clinical Biology, 9600 Zottegem, Belgium; valerie.pede@sezz.be; 39 Biogazelle NV, Diagnostic Testing, 9052 Zwijnaarde, Belgium; friedel.nollet@azsintjan.be; 40 Institute of Clinical Biology ULB-IBC, 1170 Brussels, Belgium; vincent.claes@ulb.be; 41 AZ St-Blasius Dendermonde, 9200 Dendermonde, Belgium; inge.verschraegen@azsintblasius.be; 42 CHU UCL Namur, Department of Laboratory Medicine, Molecular Diagnostics Center, 5530 Yvoir, Belgium; pierre.bogaerts@chuuclnamur.uclouvain.be; 43 General Hospital Sint-Maria Halle, 1500 Halle, Belgium; m.vangysel@sintmaria.be; 44 Practimed, 3980 Tessenderlo, Belgium; judith.leurs@practimed.be; 45 AZ Nikolaas, 9100 Sint-Niklaas, Belgium; veroniek.saegeman@aznikolaas.be; 46 Department of Microbiology and Infection Control, Universitair Ziekenhuis Brussel, Vrije Universiteit Brussel, 1090 Brussels, Belgium; oriane.soetens@uzbrussel.be; 47 Clinical Laboratory, Laboratory Medicine, AZ Delta, 8800 Roeselare, Belgium; merijn.vanhee@azsintjan.be; 48 Center for Medical Analysis, 2200 Herentals, Belgium; gilberte.schiettekatte@cma.be; 49 ZNA Middelheim, Clinical Laboratory, 2020 Antwerp, Belgium; evelyne.huyghe@zna.be; 50 General Hospital Heilig Hart Lier, 2500 Lier, Belgium; steven.martens@olvz-aalst.be; 51 AZ Sint-Maarten, Laboratory of Clinical Biology, 2800 Mechelen, Belgium; ann.lemmens@emmaus.be; 52 AZ Turnhout, 2300 Turnhout, Belgium; heleen.nailis@azturnhout.be; 53 Clinique Saint-Luc Bouge, 5004 Namur, Belgium; kim.laffineur@slbo.be; 54 Clinical Laboratory, Campus Sint-Jan, Hospital Oost-Limburg, 3600 Genk, Belgium; deborah.steensels@zol.be; 55 Clinical Laboratory, AZ Sint-Lucas Hospital, 9000 Gent, Belgium; elke.vanlaere@azstlucas.be; 56 Institute of Pathology and Genetics, 6041 Gosselies, Belgium; jeremie.gras@ipg.be; 57 Clinique Saint Pierre, Laboratory, 1340 Ottignies, Belgium; gatien.roussel@cspo.be; 58 AZ Voorkempen, Clinical Laboratory, 2390 Malle, Belgium; koenraad.gijbels@emmaus.be; 59 Clinical Laboratory, Campus Kennedylaan, AZ Groeninge, 8500 Kortrijk, Belgium; michael.boudewijns@azgroeninge.be; 60 Grand Hôpital de Charleroi, Clinical Biology and Microbiology, 6060 Gilly, Belgium; catherine.sion@ghdc.be; 61 Clinical Laboratory, Algemeen Stedelijk Ziekenhuis Aalst, 9300 Aalst, Belgium; wim.achtergael@asz.be; 62 Sint Trudo Hospital, 3800 Sint-Truiden, Belgium; wim.maurissen@stzh.be; 63 Laboratory for Medical Biology Iliano, 9070 Destelbergen, Belgium; luc.iliano@laboiliano.be; 64 CHR Verviers, Laboratory of Clinical Biology, 4800 Verviers, Belgium; marianne.chantrenne@chrverviers.be; 65 AZ Rivierenland, Campus Bornem, 2880 Bornem, Belgium; geert.vanheule@azr.be; 66 AZ Rivierenland, Campus Rumst, 2840 Rumst, Belgium; reinoud.flies@azr.be; 67 Clinical Biology Unit, Vivalia Clinique du Sud-Luxembourg, 6700 Arlon, Belgium; nicolas.hougardy@vivalia.be; 68 Clinical Laboratory, AZ Alma, 9900 Eeklo, Belgium; mario.berth@azalma.be; 69 Medical Laboratory Medina, 9880 Aalter, Belgium; vanessa.verbeke@medina.be; 70 Department of Laboratory Medicine, Campus Henri Serruys, AZ Sint-Jan Brugge, 8400 Oostende, Belgium; robin.morent@azsintjan.be; 71 Laboratory of Molecular Biology, Campus Aalst-Asse-Ninove, Onze-Lieve-Vrouwziekenhuis, 9300 Aalst, Belgium; anne.vankeerberghen@olvz-aalst.be; 72 Clinical Laboratory, Unit of Clinical Microbiology, CHU Liège, 4000 Liège, Belgium; sbontems@chuliege.be; 73 Microbiology, Algemeen Medisch Laboratorium, 2020 Antwerp, Belgium; kaat.kehoe@aml-lab.be; 74 Laboratory Medical Analysis CRI, 9052 Gent, Belgium; aschallier@cri.be; 75 Laboratory, Clinique du MontLégia, Groupe Santé CHC, 4000 Liège, Belgium; giang.ho@chc.be; 76 Clinical Laboratory, Mariaziekenhuis Noorderhart, 3900 Pelt, Belgium; kristof.bafort@noorderhart.be; 77 Laboratory for Molecular Diagnostics, Jessa Hospital, 3500 Hasselt, Belgium; marijke.raymaekers@jessazh.be; 78 Microbiology, Laboratory Somedi, 2220 Heist-op-den-Berg, Belgium; yolande.pypen@somedi.be; 79 Laboratory of Clinical Biology, Hospital Arlon—Vivalia, 6700 Arlon, Belgium; amelie.heinrichs@vivalia.be; 80 Clinical Laboratory, Ziekenhuis Geel, 2440 Geel, Belgium; wim.schuermans@ziekenhuisgeel.be; 81 Microbiology, Medilab, 9000 Gent, Belgium; dominique@medilab.be; 82 Clinical Laboratory, CHU Charleroi, 6042 Charleroi, Belgium; salaheddine.lali@chu-charleroi.be; 83 Microbiology, Algemeen Medisch Laboratorium West, 8850 Ardooie, Belgium; stefanie.drieghe@laboardooie.be; 84 Clinical Laboratory, Heilig Hart Ziekenhuis Mol, 2400 Mol, Belgium; dieter.ory@azmol.be; 85 Federal Testing Platform COVID-19, University Hospitals Antwerp, 2650 Edegem, Belgium; marie.lemercier@uza.be; 86 Federal Testing Platform COVID-19, Department of Laboratory Medicine, University Hospitals Leuven, 3000 Leuven, Belgium; kristel.vanlaethem@uzleuven.be; 87 Laboratory of Clinical and Epidemiological Virology, Department of Microbiology, Rega Institute for Medical Research, Immunology and Transplantation, KU Leuven, 3000 Leuven, Belgium; piet.maes@kuleuven.be; 88 Clinical Laboratory, AZ Vesalius Tongeren, 3700 Tongeren, Belgium; inge.thoelen@azvesalius.be; 89 Microbiology Laboratory, University Hospitals Antwerp, 2650 Edegem, Belgium; sarah.vandamme@uza.be; 90 Clinical Laboratory, Hospital Hornu Epicura, 7301 Boussu, Belgium; iqbal.mansoor@epicura.be; 91 Clinical Laboratory, AZ Klina, 2930 Brasschaat, Belgium; carl.vael@klina.be; 92 Laboratory Clinical Biology, Ziekenhuis Waregem, 8790 Waregem, Belgium; maxime.desloovere@ziekenhuiswaregem.be; 93 Microbiology, Eurofins Labo Van Poucke, 8500 Kortrijk, Belgium; k.declerck@labovanpoucke.eu; 94 Laboratory of Clinical Microbiology, Department of Microbiology, Immunology and Transplantation, KU Leuven, 3000 Leuven, Belgium

**Keywords:** SARS-CoV-2, PCR, semi-quantitative reporting, RNA copies/mL, infectivity

## Abstract

From early 2020, a high demand for SARS-CoV-2 tests was driven by several testing indications, including asymptomatic cases, resulting in the massive roll-out of PCR assays to combat the pandemic. Considering the dynamic of viral shedding during the course of infection, the demand to report cycle threshold (Ct) values rapidly emerged. As Ct values can be affected by a number of factors, we considered that harmonization of semi-quantitative PCR results across laboratories would avoid potential divergent interpretations, particularly in the absence of clinical or serological information. A proposal to harmonize reporting of test results was drafted by the National Reference Centre (NRC) UZ/KU Leuven, distinguishing four categories of positivity based on RNA copies/mL. Pre-quantified control material was shipped to 124 laboratories with instructions to setup a standard curve to define thresholds per assay. For each assay, the mean Ct value and corresponding standard deviation was calculated per target gene, for the three concentrations (10^7^, 10^5^ and 10^3^ copies/mL) that determine the classification. The results of 17 assays are summarized. This harmonization effort allowed to ensure that all Belgian laboratories would report positive PCR results in the same semi-quantitative manner to clinicians and to the national database which feeds contact tracing interventions.

## 1. Introduction

The rapid scale-up of testing capacity in combination with a testing strategy linked to contact tracing, including intense screening of asymptomatic persons, resulted in the massive roll-out of polymerase chain reaction (PCR) assays to combat the pandemic of severe acute respiratory syndrome coronavirus type 2 (SARS-CoV-2). Most PCR assays report in cycle threshold (Ct) values that can highly vary across different methods and laboratories, complemented with supply chain issues that forced many laboratories to adopt multiple SARS-CoV-2 PCR methods [1], causing the need to report in a more detailed manner than purely qualitative. Since the majority of real-time (RT-)PCR assays are not designed to report test results in a quantitative manner, it was chosen to move towards a semi-quantitative reporting approach to provide more detailed information to clinicians with respect to the stage of infection, and indirectly the potential link with infectivity. A correlation between Ct values and infectivity was aimed to make use of infectivity as a surrogate marker for transmissibility, the latter being determined by viral infectivity, contagiousness of the infected person, susceptibility of contacts and context of the contact (e.g., format, duration and location) between two persons. Nevertheless, based on a single PCR test result, it cannot be exactly judged in which stage of the infection a person is sampled, it still remains the responsibility of the clinician to interpret the test result in the context of clinical (e.g., onset of symptoms and/or date of high-risk contact) and/or serological evidence and/or the availability of (a) previous PCR test result(s).

In November 2020, a proposal to move towards a semi-quantitative way of reporting RT-PCR test results was drafted by the National Reference Centre (NRC) for Respiratory Pathogens at UZ/KU Leuven. Since no consensus on thresholds with respect to viral load and its link to contagiousness was available in literature at that time, data on which to formulate the proposal were mainly derived from in vitro studies that were characterized by an important heterogeneity of methods, study population and potential impact of pre-analytical factors on the quality of viral cell culture experiments using clinical material. The majority of these published studies report thresholds based on Ct values instead of actual viral loads in copies/mL, complicating the evaluation of cut-off values for cell culture experiments. A range of Ct values from 24 up to 35 was reported as threshold value for a so-called negative cell culture, implicating the absence of a cytopathogenic effect (CPE) when the viral material was cultivated, mainly including studies making use of clinical specimens [2,3,4,5,6,7,8,9,10,11,12,13,14], although also one epidemiological study [15]. While only two references [3,6] suggested a cut-off Ct value for culture positivity up to 30, all other studies reported Ct values in the range of 30 to 35 [2,4,5,7,8,9,10,11,12,13,14]. Overall, at the time of the literature review (November 2020), only six studies reported a threshold value for the absence of CPE based on viral load instead of Ct values, of which four studies defined this cut-off as <10^5^ copies/mL [16,17,18,19], one as <10^4^ copies/mL [20] and one as <10^6^ copies/mL [21]. Among the few epidemiological studies focusing on the association between transmission risk and viral load, Goyal et al. predicted a limited risk of transmission in the case of a viral load of <10^5^ RNA copies/mL [22]. Based on experimental data from viral cell culture experiments performed at the NRC (from a series of RT-PCR positive samples, the highest Ct value that resulted in CPE was 28.5—data not shown), a person was no longer considered to be contagious when the viral load of the respective SARS-CoV-2 infection was <10^3^ RNA copies/mL. An arbitrary threshold of >10^7^ RNA copies/mL was set to define a person as contagious with high certainty, since an overall peak viral load of 10^8^ RNA copies/mL was reported to be reached prior to or within the first days of symptoms onset [23].

After thorough discussion within the Molecular Microbiology expert group of the department Quality of Laboratories of the Belgian national public health institute Sciensano, and the risk assessment group (RAG) coordinated by Sciensano, advice to report in a semi-quantitative manner was formulated [24], dividing SARS-CoV-2 PCR positive test results into four categories according to the level of the viral load (Table 1). Of note, the category ‘weakly positive’ could certainly also reflect the early stage of a new SARS-CoV-2 infection, hence caution is warranted when interpreting a weak positive test result. For a short period in time and in a specific context of three target gene PCR assays, advice was formulated for a fifth category called ‘traces of SARS-CoV-2′ [25], defined as a viral load of <10^3^ RNA copies/mL for the nucleocapsid gene while not detected for the other two target genes, however this category is not further discussed in this manuscript. All four categories (very strongly positive to weakly positive) gave rise to contact tracing and quarantine measures, in cases where it was informed by additional clinical information. In order for an infection to be considered as old or cleared, the criteria as proposed by the RAG in June 2020 [24] still needed to be fulfilled, and when fulfilled, no isolation or contact tracing was initiated for these cases. To support national contact tracing, laboratory test results were asked to be reported in a semi-quantitative manner to the healthdata.be platform of Sciensano from April 2021 onwards [26].

To be able to report in a semi-quantitative manner, pre-quantified SARS-CoV-2 control material was prepared at the NRC for Respiratory Pathogens at UZ/KU Leuven. The control material, accompanied by a letter providing guidelines to set up a standard curve, was shipped to all laboratories in Belgium that were declared to perform SARS-CoV-2 PCR assays in a routine diagnostic setting. By doing so, the laboratories were able to define thresholds for the PCR assay(s) used in their respective laboratory, to allow the reporting of SARS-CoV-2 positive test results in the four described categories without the use of a quantitative PCR assay or the need to report Ct values. Through the detailed setup of a standard curve, the correlation between RNA copies/mL present in the control material to the Ct values or equivalent metric used for their respective assay, would be defined and could later be used to report in a semi-quantitative manner to clinicians as well as to the healthdata.be platform of Sciensano, improving infection control measures [27].

## 2. Materials and Methods

### 2.1. Preparation of SARS-CoV-2 Control Material

The NRC for Respiratory Pathogens at UZ/KU Leuven cultured a SARS-CoV-2 PCR-positive clinical sample dated 27 January 2021, on Vero E6 cells [28,29], following daily inspection to evaluate the presence of a corona-specific CPE. Once CPE was observed, a confirmation SARS-CoV-2 specific-PCR was performed, and a volume of about 340 mL of supernatant was obtained. The supernatant was heat-inactivated and centrifuged to remove leftover cell material, prior to aliquoting the large volume into Sarstedt tubes of 1 mL each and storage at −20 °C. Starting from one aliquot, dilution series (10^−1^ to 10^−7^) were prepared in triplicate to quantify the viral concentration by the use of the SARS-CoV-2 PCR assay on Alinity m (Abbott), used in routine diagnostics and set up as a quantitative assay at the NRC (for further details see next paragraph). Dilution series were prepared in Sigma MM^TM^ medium (Medical Wire) starting from 200 µL of the prepared stock. Each dilution of a total volume of 2 mL was distributed to three vials to allow triplicate testing. Whole-genome sequencing (WGS) according to a previous published protocol [30,31] was performed on the supernatant to allow classification and detailed mapping of the mutations and deletions across the SARS-CoV-2 genome.

Quantitative determination of the viral load of the stock material was possible due to the earlier setup of a standard curve for the Alinity m SARS-CoV-2 assay, using the SARS-CoV-2 molecular control SCV2MQC01-B (Qnostics, Glasgow, Scotland, UK), a commercially available control with known genome equivalents. In April 2020, this Qnostics control was used at first to set up a standard curve for an early Belgian SARS-CoV-2 isolate, quantifying at that time the viral load of the cell culture using a lab-developed test (LDT) on QuantStudio Dx [32]. The April 2020 quantified SARS-CoV-2 stock was further used to prepare dilution series in September 2020 to set up a standard curve for the Alinity m SARS-CoV-2 assay, implementing it as a quantitative assay for use in routine diagnostics at the NRC UZ/KU Leuven. While the concentration of the SARS-CoV-2 control material should be expressed as ‘genome equivalents’ due to its calibration towards the Qnostics standard, for a broad audience understanding, throughout the manuscript the concentration is expressed as ‘copies per mL’.

### 2.2. Distribution of SARS-CoV-2 Control Material and Setup of Standard Curves

The pre-quantified SARS-CoV-2 material was shipped on dry ice to 124 recognized COVID-19 testing laboratories across Belgium [32,33], using direct transport. The laboratories were asked to retain the storage condition of −20 °C until the time of analysis to prevent additional freeze-thaw cycles. The stock was accompanied by a letter in which recommendations were formulated with respect to the preparation and testing of dilutions in order to set up a standard curve in a standardized manner. Dilutions of 10^8^ to 10 copies/mL, complemented with a negative control, were proposed to evaluate the possibility to report test results in a semi-quantitative way. An important condition that needs to be met when reporting in this way is the possibility to be able to thoroughly evaluate the test result of the internal control (IC) to account for underestimation of the viral load category. Each dilution was recommended to be tested in triplicate to allow calculating the average of these measurements for each gene that is targeted in the respective PCR assay. The advice was given to implement the average thresholds for the gene that was found to be associated with the highest sensitivity complemented with the knowledge on circulating variants at the time.

### 2.3. Data Processing: Data Requirements

The laboratories that participated in this harmonization effort were asked to report results of the standard curve(s) they set up, to the NRC, in order to process the data and to prepare a summary comparing the different PCR assays in use in Belgium. Despite this summary, it was advised to implement the cut-off values as determined by the standard curve setup in the respective laboratory, following procedures as validated in the routine diagnostic flow, rather than implementing standard thresholds resulting from data of multiple laboratories. Solely, the PCR assay that was used to set up the standard curve was considered when processing the results, not the sample preparation specifics, extraction method nor the specific equipment used to perform the PCR.

Since a large number of laboratories participated to this harmonization effort, criteria were defined that needed to be fulfilled in order for the respective results of the standard curve to be included in the downstream data analysis. At first, the standard curve of a particular PCR assay needed to be set up by a minimum of two laboratories to make a comparison possible. This automatically entailed that LDT assays were not considered. Secondly, data were only used when each dilution series was measured in duplicate or triplicate. As a third criterion, only results derived from a standard curve with a R^2^ coefficient of ≥0.95 were considered. Finally, during data analysis, the results of a laboratory could still be excluded when inclusion of the respective results caused an increase in the standard deviation (SD) of 1 or more, as such a standard curve was considered to be an outlier compared to the results shared by other participating laboratories.

### 2.4. Data Processing: Approach for Each PCR Assay

For each individual PCR assay, the mean Ct value or equivalent metric, with the corresponding SD, was calculated per target gene of the respective assay, based on the results derived from the standard curve set up by each individual laboratory. The mean Ct value was calculated for the three concentrations (10^7^, 10^5^ and 10^3^ RNA copies/mL) that determine the classification of the four categories. In case the SD exceeded 2, the summary of the respective RT-PCR assay was not included in the further data analysis since the dispersion of the results that different laboratories submitted, was simply too high to rely on the respective results. SD values exceeding 1 were included, however noted to carefully interpret the average values since results across the laboratories appeared to be scattered.

For most PCR assays, more than one SARS-CoV-2 gene is targeted, hence why results were analyzed for the different target genes and advice was provided on the gene to be used in the context of semi-quantitative reporting. The choice of this gene was based on sensitivity, accuracy and reproducibility complemented with the knowledge at that time on circulating SARS-CoV-2 variants and their potential impact on PCR performance.

## 3. Results

### 3.1. Preparation of the SARS-CoV-2 Control Material

Triplicate testing of dilution series (10^−1^ to 10^−7^) on Alinity m (Abbott) were used to calculate the viral load of the SARS-CoV-2 control material, as detailed in the Section 2. An average viral load of 9.04 log copies/mL (or 1.10 × 10^9^ copies/mL or in fact 1.10 × 10^9^ genome equivalents) was obtained for the stock called ‘SARS-CoV-2 strain BetaCoV/Belgium/GHB-0127/2021’. WGS classified the strain to the type 20A according to Nextclade [34], and to the Pangolin [35] lineage B.1.160. Furthermore, the following amino acid changes were detected across the full-length genome (compared to the Wuhan reference NC_0.45512.2): N-gene: M234I and A376T; ORF1a: M3087I; ORF1b: A176S, P314L, V767L, K1141R and E1184D; ORF3a: Q57H; and the S-gene: S477N and D614G.

### 3.2. Distribution of the SARS-CoV-2 Control Material to the Testing Laboratories in Belgium

**Figure 1:**
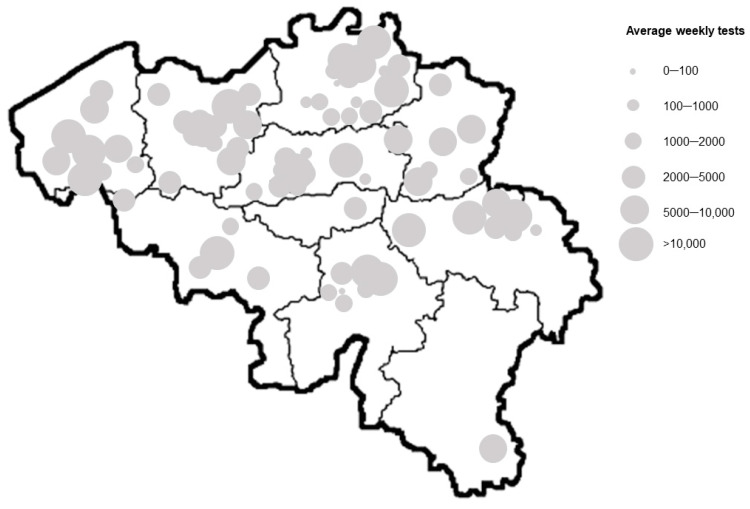
Geographic distribution of COVID-19 testing laboratories in Belgium that shared results of the standard curve set up with the distributed control material, with the NRC UZ/KU Leuven. The location of the laboratory is indicated by a dot of which the size is representative of the average PCR assays conducted on a weekly basis (using HealthData test results of weeks 47–49 of year 2021). For a limited number of laboratories, no weekly test data were available or test results were embedded within one of the multiple sites associated to the laboratory. Due to the close proximity of some laboratories and the high number of participating laboratories, dots are overlapping, no longer clearly visualizing the location of each participating laboratory.

Of the 124 recognized COVID-19 testing laboratories to which the control material was shipped, 91 laboratories, geographically dispersed across the country (Figure 1), reported results of the respective standard curve(s) to the NRC. Since numerous laboratories have adopted more than one PCR assay in routine diagnostics, this resulted in a total number of 172 evaluations spanning the wide arsenal of 41 PCR assays used across Belgium. When applying the inclusion criteria as defined in the Section 2, the results of 53 laboratories for 17 different RT-PCR assays were considered for downstream analysis. While 73.4% of the laboratories (91/124) were represented in this study, considering the number of SARS-CoV-2 PCR assays that these 91 participating laboratories perform on a weekly basis (Figure 1) compared to the overall number of tests conducted on a national level (source: HealthData Sciensano), 86.1% of the testing activity of Belgium is estimated to be covered (for weeks 47–49 of the year 2021—at that time a stable and representative dataflow to healthdata.be was in place [26]: on average 539.005 out of 626.357 tests performed by the participating laboratories). As clearly visible on the map of Belgium (Figure 1), the majority of participating laboratories (63.7% or 58/91) are located in the northern region of Belgium, Flanders. In the southern part of Belgium, Wallonia, 27.5% (25/91) of the participating laboratories are located, while 8.8% or 8 laboratories are in the Brussels capital region.

### 3.3. The SARS-CoV-2 Nucleocapsid Gene Is Most Often Targeted in PCR Assays Used in Routine Diagnostics in Belgium

Among the 41 PCR assays for which results were shared, the following five assays were used by more than 10 laboratories in Belgium: Allplex SARS-CoV-2 assay or Allplex SARS-CoV-2/FluA/FluB/RSV Assay (Seegene, 25 laboratories), Xpert^®^ Xpress SARS-CoV-2 or Xpert^®^ Xpress SARS-CoV-2/Flu/RSV (Cepheid, 24 laboratories), TaqPath COVID-19 CE-IVD RT-PCR Kit (Thermo Fisher Scientific, 22 laboratories), ARIES^®^ SARS-CoV-2 Assay (RUO, LDT or CE-IVD) (Luminex, 12 laboratories) and GeneFinderTM COVID-19 Plus RealAmp kit (Osang Healthcare, 11 laboratories) (Table 2). The 17 different PCR assays that fulfilled the inclusion criteria (as detailed in the Methods) are highlighted (*) in Table 2.

Since more than one SARS-CoV-2 gene is targeted for most PCR assays, results were analyzed for all different genes targeted in each respective PCR assay. Based on sensitivity, accuracy and reproducibility complemented with knowledge at the time of the analysis on circulating SARS-CoV-2 variants and their potential impact on the performance of RT-PCR assays, advice was provided on which gene to use in the context of semi-quantitative reporting. When considering only the assays that were used by more than one Belgian laboratory, but weighting the number of laboratories that have implemented each particular SARS-CoV-2 assay, the following order of SARS-CoV-2 genes that are most often targeted by PCR assays used in routine diagnostics in Belgium was obtained: nucleocapsid (N, *n* = 127), envelope (E, *n* = 83), RNA-dependent RNA polymerase (RdRp, *n* = 60), spike (S, *n* = 56), open-reading frame 1ab (ORF1ab, *n* = 42), ORF8 (*n* = 4) and non-structural protein 2 (Nsp2, *n* = 2).

### 3.4. The Difference in Cycle Threshold Values between the Wide Arsenal of SARS-CoV-2 Assays

After excluding 21 PCR assays for which only one laboratory had shared data (including LDT assays) and the laboratories that only measured the dilution series once, the range of R^2^ coefficient values was 0.945–0.999 with a mean of 0.995, considering all targeted SARS-CoV-2 genes. After excluding results from a standard curve with an R^2^ coefficient of <0.95 and those with a high mean SD value (see Table 2—an additional three assays), a total of 17 PCR assays remained for further downstream analysis.

The mean Ct value or equivalent metric, with the corresponding SD, was calculated per target gene for these 17 PCR assays, based on the results provided by the participating laboratories (Table 3). The mean Ct value was calculated for the three concentrations (10^7^, 10^5^ and 10^3^ RNA copies/mL) that determine the classification of the four categories. When more than one SARS-CoV-2 gene was targeted, a gene for semi-quantitative reporting was proposed as discussed earlier.

Initially all categories were interpreted as SARS-CoV-2 positive test results, followed by contact tracing and giving rise to equal quarantine and isolation measures, unless information was available to consider an infection as old or cleared [24]. More recently, in January 2022, due to the high number of infections being reported on a daily basis in the context of the Omicron wave, contact tracing scripts started to prioritize the categories ‘very strongly positive’ and ‘strongly positive’ in the region of Flanders to rapidly and more efficiently attempt to prevent further onward transmission chains. Since 17 March 2022, systematic contact tracing is no longer in place on a national level.

## 4. Discussion

### 4.1. Harmonized Semi-Quantitative Reporting of 85% of the PCR Tests in Belgium

The rapid scale-up of testing capacity, combined with a testing strategy linked to contact tracing, including asymptomatic screenings, resulted in the massive role of PCR assays to combat the SARS-CoV-2 pandemic. As Ct values can highly vary across methods and even between laboratories using the same PCR assay, a semi-quantitative approach based on RNA copies/mL was chosen to provide more details to clinicians with respect to the stage of infection, although additional clinical and/or serological evidence remains key for interpretation. Out of 124 laboratories performing daily routine diagnostic tests for SARS-CoV-2, 91 participated to this study, enabling a considerable impact of the study’s outcome on the national testing practice. Considering the number of PCR assays that the 91 participating laboratories conduct on a weekly basis, it was estimated that over 85% of the testing activity of Belgium is covered. Thanks to the harmonization effort initiated by the NRC UZ/KU Leuven distinguishing four categories of SARS-CoV-2 positivity (very strongly positive to weakly positive), many Belgian laboratories currently report in a similar semi-quantitative manner to clinicians and to healthdata.be, improving infection control measures. Providing more detailed information on the stage of infection by using the proposed semi-quantitative categories, moving beyond reporting of a purely qualitative test result, allows clinicians and the national contact tracing services to rapidly identify recent infections and subsequentially to embank superspreading transmission events. Moreover, it facilitates the follow-up of long-term SARS-CoV-2 positive patients and the potential impact of such repeated positive PCR results on the clinical management of these individuals as well as its impact on the overall hospital hygiene management. Nevertheless, as previously noted, a weakly positive test result certainly can also reflect the early stage of a new SARS-CoV-2 infection, next to an old, cleared infection, warranting the need to carefully interpret such PCR test results.

### 4.2. Limitations When Reporting Test Results Based on Ct Values

Despite the attempt to harmonize the reporting of SARS-CoV-2 PCR positive test results at a national level, we need to recognize the remaining limitations when reporting laboratory test results in the form of SARS-CoV-2 positivity categories as measured in the large majority of participating laboratories based on Ct values [36]. To summarize the mean Ct value (or equivalent metric) and its associated SD, only the PCR assay used at a participating laboratory was considered, not the sample preparation specifics, extraction method nor the specific equipment used to perform the PCR. We hypothesize that among the 17 PCR assays analyzed in detail, those with higher SD values compared to the other assays analyzed, were most likely preceded by a variety of different extraction methods, giving rise to the reported higher variability between mean Ct values. While we do not have detailed information on the extraction methods used by the participating laboratories, this hypothesis is supported by the fact that we do observe a lower variability for the sample-to-result systems (e.g., Abbott RealTime and Alinity m) in which the extraction procedure is included in the same system in comparison to open systems (e.g., PerkinElmer RT-PCR assay) for which extraction and PCR are two separate assays. Large intra- and inter-assay variability has been reported for numerous SARS-CoV-2 assays, respectively up to 10 Ct units [37] and even up to 20 Ct units between different assays [38]. During this harmonization effort, for the viral concentration of 10^7^ RNA copies/mL a wide variety of Ct values was observed, ranging from 6.2 to 20.3, while for the categories determined by a viral load of respectively 10^5^ RNA copies/mL and 10^3^ RNA copies/mL, the following ranges were reported: Ct 12.7 to 27.4, and Ct 19.1 to 34.4. The lowest Ct values were observed for the PCR assay Abbott RealTime SARS-CoV-2, substantially lower compared to the Ct values obtained for the other 16 PCR assays included in the analysis. In at least two other studies, lower Ct values have also been reported for this assay [39,40] related to the first ten PCR cycles being unread. The highest Ct values were reported for the N-gene target of the PCR assay Aries SARS-CoV-2 RUO, however not defined as actual outlier. For samples with a low viral load (i.e., 10^3^ RNA copies/mL), a higher variability was observed and is to be expected compared to samples with a higher viral load (10^5^ and 10^7^ RNA copies/mL), increasing the uncertainty range for the categories of moderate positive and weakly positive compared to the categories very strongly and strongly positive, possibly impacting the interpretation of these test results. Of note, when interpreting the results of this study, correct interpretation is key since, in this study, no absolute reference material was used to calibrate the SARS-CoV-2 control material. The standard Qnostics, expressed in genome equivalents instead of RNA copies/mL, was used since, at the time of initial calibration of the standard curve performed at the NRC UZ/KU Leuven, no absolute reference material was available. While it is more correct to express the viral concentration of the control material in genome equivalents, this term is not widely used nor easily interpretable. Currently, a WHO International Standard (NIBSC code: 20/146) is available [41], characterized with a viral concentration of 7.70 log10 IU/mL.

The sampling method, including the execution, type of sampling material, sampling site and virus transport medium, have all been reported to have an important impact on the outcome of PCR test results [42,43]. In the context of this harmonization effort, these factors have not been considered, however it is well documented that they can impact Ct values to an significant extent [44,45]. Similar to the extraction method that precedes the PCR assay, no complete information with respect to the sampling matrices analyzed in the different participating laboratories was available, although it is expected that an notable difference would be observed. Furthermore, an important condition that needs to be met when reporting in a semi-quantitative manner, is the possibility to be able to thoroughly evaluate the test result of the IC to account for false negative test results or the underestimation of the viral load category due to increased Ct values. In order to account for inhibition, each PCR assay would ideally need to include an exogen IC for which thresholds were predefined to allow evaluation of acceptance criteria prior to reporting Ct values of SARS-CoV-2. Assays including only endogen IC (such as human genes) provide an indication for a correct sampling procedure, however the results of such ICs are highly variable and, therefore, are not to be used to detect partial inhibition.

### 4.3. Impact of the Target Composition of PCR Assays on SARS-CoV-2 Genomic Surveillance

When weighting the number of laboratories that have implemented each particular SARS-CoV-2 assay, the nucleocapsid was identified to be the gene most often targeted by PCR assays used in routine diagnostics in Belgium. While the detection of viral RNA by PCR assays is the gold-standard method for COVID-19 diagnosis, dropouts or shifts in Ct values of the genes targeted in the PCR assay can serve as indicators for the presence of particular variants of concern (VOC). Evaluating mismatches between the set of primers and probes used during RT-PCR and the SARS-CoV-2 genomes of a variety of VOCs, is important to assess the impact of these VOCs on the performance of routine diagnostic tests [46]. Various assays have been reported to be associated with either a dropout or shift in Ct values of a specific target gene compared to the Ct values of the other target gene(s), in the context of the presence of a particular VOC, e.g., higher Ct value for the N-gene compared to S and RdRp for the assay Allplex SARS-CoV-2/FluA/FluB/RSV in the context of the Alpha variant [47]. While the spike protein was ranked only fourth in the list of genes most often targeted by PCR, throughout the pandemic this gene has proven multiple times to be useful in the context of near-realtime tracking and surveillance of SARS-CoV-2 variants [48,49,50]. Since the emergence of Alpha, the detection of the spike protein was closely followed as a marker of this variant when using the TaqPath COVID-19 CE-IVD RT-PCR assay. Both VOCs Alpha and Omicron BA.1 have been identified to be associated with an S-gene target failure (SGTF), which is defined as the detection of both target genes N and ORF1ab (with a sufficient high viral load) while the S-gene is not detected. During the emergence of both variants Alpha and Omicron BA.1, other variants were circulating, stressing the added value of following SGTF, dropouts or shifts in Ct values of target genes in routine diagnostics. These changes allowed the early detection of the emergence of a new variant in the general population, next to the roll-out of a strong genomic surveillance approach by WGS, however the turn-around-time is substantially longer compared to PCR.

### 4.4. Interpretation of SARS-CoV-2 PCR Test Results

Overall, the large variability in Ct values reported between PCR assays and even between laboratories using the same SARS-CoV-2 assay advocates to not simply report pure Ct values. By implementing semi-quantitative categories of SARS-CoV-2 positivity, a compromise was initiated between the request of clinicians to detail SARS-CoV-2 test results to a maximum and a correct laboratory estimate of viral load. Especially laboratories that have implemented several PCR assays in parallel, due to the shortage of reagents and the need to upscale their testing capacity, should be cautious when directly reporting Ct values to clinicians. We strongly encourage these laboratories to invest into harmonization of PCR test results through the use of standards to control the process or through the implementation of the proposed SARS-CoV-2 positivity categories to balance the estimation of viral load based on Ct values. Although through this harmonization effort a summary of mean Ct values and associated SD has become available for the 17 most used PCR assays in Belgium, it is of particular importance that laboratories should never implement these thresholds as such but should only rely on standard curve data obtained in their own laboratory setting, following the standard operating procedures in place. Furthermore, standard curves have been initiated for the period February to March 2021, using pre-quantified control material dating from January 2021, after which additional PCR assays have been implemented in some laboratories and followed by the emergence of numerous SARS-CoV-2 VOCs and variants of interest, of which some have been reported to impact PCR performance [47,48,49,50]. The target gene proposed per PCR assay to use in the context of semi-quantitative reporting may, therefore, change over time, in the case that the emergence of a new circulating variant that may potentially cause either a shift in Ct values between SARS-CoV-2 genes or a complete dropout of a gene when a specific mutation or deletion (e.g., 69–70 deletion in the spike protein) [51] arises. However, due to the implementation of multitarget PCR assays in Belgium, in general targeting of highly conserved regions of the SARS-CoV-2 genome, it is not expected that the emergence of new variants will highly impact the overall diagnostic PCR performance and result into false-negative test results. Nevertheless, the virus will remain to continuously evolve, warranting the need to revise the use of specific genes in the context of reporting.

The indirect link between the SARS-CoV-2 PCR positive test result and the infectivity needs to be interpreted with caution. A positive PCR test does not necessarily entail the presence of viable virus [52], as some patients can be infectious before a PCR test turns out be positive. However, the turn-around-time to perform viral culture experiments does not match the current urgency in which SARS-CoV-2 test results are expected to be reported. In order to efficiently initiate isolation and quarantine procedures to limit onwards transmission, rapid interpretation of SARS-CoV-2 PCR test results is warranted. At the time of the initiation of this harmonization effort, numerous publications already reported the link between disease stage and transmissibility. Overall, infectivity beyond day 10 of symptom onset was rarely reported, although a number of publications describe the persistence of culturable virus beyond day 10 from symptom onset [8,11,12,14,22,53,54,55], however, these all related to individual or immunocompromised cases.

## 5. Conclusions

Due to the massive roll-out of PCR assays, including in the context of intense screening of asymptomatic persons, the demand to report Ct values rapidly emerged. Since Ct values can be affected by a number of factors and can highly vary between assays and laboratories, it was decided to move towards a semi-quantitative approach to provide more details to clinicians with respect to the stage of infection, and indirectly the potential link with infectivity. Thanks to this harmonization effort, many Belgian laboratories currently report SARS-CoV-2 positive PCR results in the same semi-quantitative manner, distinguishing four categories of positivity based on RNA copies/mL, to clinicians and to healthdata.be, improving infection control measures.

## Figures and Tables

**Table 1:** Proposed interpretation and reporting scheme for SARS-CoV-2 PCR results [24]: a semi-quantitative reporting approach of PCR test results into four categories of SARS-CoV-2 positivity.

Category of SARS-CoV-2 Positivity	SARS-CoV-2 Viral Load (RNA Copies/mL)	Interpretation with Respect to Infectivity
Very strongly positive	≥10^7^	patient is contagious
Strongly positive	≥10^5^–<10^7^	patient is probably contagious
Moderate positive	≥10^3^–<10^5^	patient is potentially contagious, unless there is clinical and/or serological evidence of an old, cleared infection
Weakly positive	<10^3^	patient is probably not or no longer contagious if there is also clinical and/or serological evidence of an old, cleared infection

**Table 2:** Overview of PCR assays used by laboratories in Belgium that were declared to perform SARS-CoV-2 testing in a routine diagnostic setting. Only PCR assays for which the results of the setup standard curve were shared with the NRC, were considered. The number of laboratories that use each PCR assay is listed, as well as the targeted SARS-CoV-2 genes. The PCR assays are listed in alphabetical order.

SARS-CoV-2 PCR Assay	Number of Laboratories	Targeted SARS-CoV-2 Genes
Abbott Realtime SARS-CoV-2 assay (m2000) *	3	RdRp and N
Alinity m SARS-CoV-2 assay *	6	RdRp and N
Allplex SARS-CoV-2 assay *	21	E, RdRp, S and N
Allplex SARS-CoV-2/FluA/FluB/RSV assay *	8	S, RdRp and N
Aries SARS-CoV-2 LDT	4	E
Aries SARS-CoV-2 RUO *	10	ORF1ab and N
BD SARS-CoV-2 for BD Max *	3	N1 and N2
CE-IVD Perkin Elmer SARS-CoV-2 RT-qPCR *	2	ORF1ab and N
Cobas SARS-CoV-2 *	4	ORF1ab and E
GeneFinderTM COVID-19 Plus RealAmp kit *	12	RdRp, E and N
Ingenius	2	RdRp, E and N
Liat Cobas SARS-CoV-2 and influenza A/B ^1^	4	RdRp and N
LightMix Modular SARS and Wuhan CoV E-gene kit ^2^	5	E
N1 CDC PCR	2	N1
NeuMoDx SARS-CoV-2 *	2	N and Nsp2
Powerchek SARS-CoV-2 Real-time PCR kit ^1^	2	ORF1ab and E
R-DiaSARS-CoV-2 *	3	E
RealStar SARS-CoV-2 RT-PCR kit 1.0 *	5	E and S
SARS-CoV-2 ELITe MGB kit *	2	RdRp and ORF8
SARS-CoV-2 plus ELITe MGB *	2	RdRp, ORF8
SARS-CoV-2 N1 + N2 Qiagen ^2^	3	N1 and N2
TaqPath COVID-19 CE-IVD RT-PCR kit *	22	N, S and ORF1ab
Viasure SARS-CoV-2 RT PCR ^1^	2	ORF1ab and N
Xpert Xpress SARS-CoV-2 *	14	N2 and E
Xpert Xpress SARS-CoV-2/Flu/RSV *	11	N2 and E

Abbreviations: CE-IVD (CE-in vitro diagnostic), LDT (laboratory developed test), RUO (research use only). Assays that are used by only one Belgian laboratory in this harmonization effort, and hence are not listed in the Table: Allplex 2019-nCoV assay, Altostar SARS-CoV-2 RT-PCR Kit 1.5, Aries SARS-CoV-2 CE-IVD, Charité E gene assay, Cobas SARS-CoV-2 LDT, Diagenode Diagnostics RUO, FLOW, GenomEra SARS-CoV-2, Simplexa COVID-19 Direct, TAC-Respi TLDA-Respi array, UC on Cobas 6800, Viasure SARS-CoV-2 N1 + N2, VitaPCR Credo and several homemade/LDT PCRs. The assays that fulfilled the inclusion criteria for downstream analysis are highlighted with an asterix (*). Reason for exclusion: ^1^ Although more than one laboratory shared data, less than two laboratories measured each dilution series two or three times. ^2^ The standard deviation of the results contributed by the different laboratories exceeds 2.

**Table 3:** Overview of mean cycle threshold (Ct) values with corresponding standard deviation (SD) for the 17 PCR assays that meet the inclusion criteria for downstream analysis, based on the data provided by the laboratories that were included (see Section 2). Mean results should be carefully interpreted, especially when SD values exceed 1 (highlighted in red). The proposed target gene to use for semi-quantitative reporting is underlined in case applicable.

PCR Assay	Included (Contributed) Laboratories	SARS-CoV-2 Gene	Mean Ct Value or Equivalent Metric (SD)		
			10^7^ RNA Copies/mL *	10^5^ RNA Copies/mL *	10^3^ RNA Copies/mL *
Abbott RealTime SARS-CoV-2	3 (3)	RdRp/N	6.2 (0.34)	12.7 (0.37)	19.1 (0.50)
Alinity m SARS-CoV-2	6 (6)	RdRp/N	15.0 (0.65)	21.9 (0.68)	28.9 (0.75)
Allplex SARS-CoV-2	15 (19)	E	17.0 (0.49)	24.3 (0.72)	31.5 (1.02)
		RdRp/S	17.9 (0.76)	25.1 (0.96)	32.2 (1.28)
		N	17.9 (0.67)	24.9 (0.80)	31.9 (1.04)
Allplex SARS-CoV-2/FluA/FluB/RSV	7 (8)	S	15.7 (0.44)	22.6 (0.55)	29.6 (0.82)
		RdRp	15.3 (0.47)	22.5 (0.63)	29.7 (0.89)
		N	15.4 (0.54)	22.1 (0.71)	28.8 (0.99)
Aries SARS-CoV-2 RUO	7 (9)	N	20.3 (0.68)	27.4 (0.78)	34.4 (0.96)
		ORF1ab	18.3 (0.54)	25.3 (0.73)	32.4 (1.06)
BioGX SARS-CoV-2 BD MAX	3 (3)	N1	16.4 (0.71)	23.4 (0.56)	30.4 (0.42)
		N2	16.8 (0.97)	23.8 (0.99)	30.9 (1.03)
Cobas SARS-CoV-2	4 (4)	ORF1ab	17.3 (0.50)	23.6 (0.52)	29.8 (0.56)
		E	17.6 (0.50)	24.0 (0.51)	30.4 (0.55)
GeneFinder COVID-19 Plus RealAmp	6 (13)	E	18.9 (0.37)	26.1 (0.61)	33.3 (0.85)
		RdRp	18.6 (0.65)	25.3 (0.74)	32.1 (0.88)
		N	19.4 (0.99)	26.2 (1.00)	33.0 (1.06)
NeuMoDx SARS-CoV-2	2 (2)	N	15.7 (0.09)	22.6 (0.10)	29.4 (0.29)
		Nsp2	16.4 (0.01)	23.3 (0.31)	30.2 (0.62)
PerkinElmer SARS-CoV-2 RT-PCR	2 (2)	N	18.8 (2.34)	25.1 (1.89)	31.5 (1.45)
		ORF1ab	16.4 (1.82)	22.7 (1.63)	28.9 (1.44)
R-DiaSARS-CoV-2	3 (3)	E	17.6 (0.39)	23.9 (0.44)	30.3 (0.68)
RealStar SARS-CoV-2 RT-PCR kit 1.0	2 (4)	S	16.3 (1.38)	23.1 (1.69)	30.0 (2.00)
		E	19.1 (1.10)	25.6 (1.49)	32.2 (1.87)
SARS-CoV-2 ELITe MGB	2 (2)	RdRp	18.8 (0.84)	25.4 (0.84)	32.0 (0.83)
		ORF8	17.8 (0.86)	24.6 (0.94)	31.3 (1.02)
SARS-CoV-2 plus ELITe MGB	2 (2)	RdRp/ORF8	16.8 (0.66)	23.8 (0.71)	30.9 (0.76)
TaqPath COVID-19 CE-IVD RT-PCR	17 (21)	N	14.5 (0.69)	21.3 (0.78)	28.1 (0.95)
		ORF1ab	14.1 (0.71)	20.9 (0.75)	27.8 (0.95)
		S	14.1 (0.70)	20.9 (0.74)	27.8 (0.92)
Xpert Xpress SARS-CoV-2	7 (13)	N2	19.1 (0.48)	26.2 (0.69)	33.2 (1.04)
		E	16.7 (0.49)	23.4 (0.51)	30.1 (0.54)
Xpert Xpress SARS-CoV-2/Flu/RSV	9 (11)	N2/E	16.5 (0.57)	23.2 (0.92)	29.9 (1.35)

* Due to calibration of the SARS-CoV-2 control material towards the commercially available control of Qnostics, copies per mL are, in fact, expressed as genome equivalents.

## Data Availability

Not applicable.

## References

[1] Mardian Y., Kosasih H., Karyana M., Neal A., Lau C.-Y. (2021). Review of current COVID-19 diagnostics and opportunities for further development. Front Med..

[2] La Scola B., Le Bideau M., Andreani J., Hoang V.T., Grimaldier C., Colson P., Gautret P., Raoult D. (2020). Viral RNA load as determined by cell culture as a management tool for discharge of SARS-CoV-2 patients from infectious disease wards. Eur. J. Clin. Microbiol..

[3] Bullard J., Dust K., Funk D., Strong J.E., Alexander D., Garnett L., Boodman C., Bello A., Hedley A., Schiffman Z. (2020). Predicting infectious severe acute respiratory syndrome coronavirus 2 from diagnostic samples. Clin. Infect. Dis..

[4] Jefferson T., Spencer E.A., Brassey J., Heneghan C. (2020). Viral cultures for coronavirus disease 2019 infectivity assessment: A systematic review. Clin. Infect. Dis..

[5] Arons M.M., Hatfield K.M., Reddy S.C., Kimball A., James A., Jacobs J.R., Taylor J., Spicer K., Bardossy A.C., Oakley L.P. (2020). Presymptomatic SARS-CoV-2 infections and transmission in a skilled nursing facility. N. Engl. J. Med..

[6] Brown C.S., Clare K., Chand M., Andrews J., Auckland C., Beshir S., Choudhry S., Davies K., Freeman J., Gallini A. (2020). Snapshot PCR surveillance for SARS-CoV-2 in hospital staff in England. J. Infect..

[7] Folgueira M.D., Luczkowiak J., Lasala F., Pérez-Rivilla A., Delgado R. (2021). Prolonged SARS-CoV-2 cell culture replication in respiratory samples from patients with severe COVID-19. Clin. Microbiol. Infect..

[8] Basile K., McPhie K., Carter I., Alderson S., Rahman H., Donovan L., Kumar S., Tran T., Ko D., Sivaruban T. (2020). Cell-based Culture Informs Infectivity and Safe De-Isolation Assessments in Patients with Coronavirus Disease 2019. Clin. Infect. Dis..

[9] Singanayagam A., Patel M., Charlett A., Bernal J.L., Saliba V., Ellis J., Ladhani S., Zambon M., Gopal R. (2020). Duration of infectiousness and correlation with RT-PCR cycle threshold values in cases of COVID-19, England, January to May 2020. Eurosurveillance.

[10] Gniazdowski V., Paul Morris C., Wohl S., Mehoke T., Ramakrishnan S., Thielen P., Powell H., Smith B., Armstrong D.T., Herrera M. (2021). Repeated Coronavirus Disease 2019 Molecular Testing: Correlation of Severe Acute Respiratory Syndrome Coronavirus 2 Culture With Molecular Assays and Cycle Thresholds. Clin. Infect. Dis..

[11] Young B.E., Ong S.W.X., Ng L.F.P., Anderson D.E., Chia W.N., Chia P.Y., Ang L.W., Mak T.-M., Kalimuddin S., Chai L.Y.A. (2021). Viral Dynamics and Immune Correlates of Coronavirus Disease 2019 (COVID-19) Severity. Clin. Infect. Dis..

[12] Rhee C., Kanjilal S., Baker M., Klompas M. (2020). Duration of Severe Acute Respiratory Syndrome Coronavirus 2 (SARS-CoV-2) Infectivity: When Is It Safe to Discontinue Isolation?. Clin. Infect. Dis..

[13] Ladhani S.N., Chow J.Y., Janarthanan R., Fok J., Crawley-Boevey E., Vusirikala A., Fernandez E., Perez M.S., Tang S., Dun-Campbell K. (2020). Investigation of SARS-CoV-2 outbreaks in six care homes in London, April 2020. EClinicalMedicine.

[14] Jaafar R., Aherfi S., Wurtz N., Grimaldier C., Van Hoang T., Colson P., Raoult D., La Scola B. (2020). Correlation between 3790 Quantitative Polymerase Chain Reaction–Positives Samples and Positive Cell Cultures, Including 1941 Severe Acute Respiratory Syndrome Coronavirus 2 Isolates. Clin. Infect. Dis..

[15] Chen F., Fu D., Yang Q., Geng Z., Xia J., Wang Z., Wang L. (2020). Low transmission risk of 9 asymptomatic carriers tested positive for both SARS-CoV-2 nucleic acid and serum IgG. J. Infect..

[16] Wölfel R., Corman V.M., Guggemos W., Seilmaier M., Zange S., Müller M.A., Niemeyer D., Jones T.C., Vollmar P., Rothe C. (2020). Virological assessment of hospitalized patients with COVID-2019. Nature.

[17] Huang C.-G., Lee K.-M., Hsiao M.-J., Yang S.-L., Huang P.-N., Gong Y.-N., Hsieh T.-H., Huang P.-W., Lin Y.-J., Liu Y.-C. (2020). Culture-Based Virus Isolation To Evaluate Potential Infectivity of Clinical Specimens Tested for COVID-19. J. Clin. Microbiol..

[18] Perera R.A., Tso E., Tsang O.T., Tsang D.N., Fung K., Leung Y.W., Chin A.W., Chu D.K., Cheng S.M., Poon L.L. (2020). SARS-CoV-2 Virus Culture and Subgenomic RNA for Respiratory Specimens from Patients with Mild Coronavirus Disease. Emerg. Infect. Dis..

[19] Pekosz A., Parvu V., Li M., Andrews J.C., Manabe Y.C., Kodsi S., Gary D.S., Roger-Dalbert C., Leitch J., Cooper C.K. (2021). Antigen-Based Testing but Not Real-Time Polymerase Chain Reaction Correlates With Severe Acute Respiratory Syndrome Coronavirus 2 Viral Culture. Clin. Infect. Dis..

[20] L’Huillier A.G., Torriani G., Pigny F., Kaiser L., Eckerle I. (2020). Culture-Competent SARS-CoV-2 in Nasopharynx of Symptomatic Neonates, Children, and Adolescents. Emerg. Infect. Dis..

[21] Van Kampen J.J.A., Van De Vijver D.A.M.C., Fraaij P.L.A., Haagmans B.L., Lamers M.M., Okba N., Van Den Akker J.P.C., Endeman H., Gommers D.A.M.P.J., Cornelissen J.J. (2021). Duration and key determinants of infectious virus shedding in hospitalized patients with coronavirus disease-2019 (COVID-19). Nat. Commun..

[22] Goyal A., Reeves D.B., Cardozo-Ojeda E.F., Schiffer J.T., Mayer B.T. (2021). Wrong person, place and time: Viral load and contact network structure predict SARS-CoV-2 transmission and super-spreading events. Elife.

[23] Jones T.C., Biele G., Mühlemann B., Veith T., Schneider J., Beheim-Schwarzbach J., Bleicker T., Tesch J., Schmidt M.L., Sander L.E. (2021). Estimating infectiousness throughout SARS-CoV-2 infection course. Science.

[24] RAG Meeting 08/12/2020: Interpretation and Reporting of SARS-CoV-2 PCR Results. https://covid-19.sciensano.be/sites/default/files/Covid19/20201208_Advice%20RAG%20Interpretation%20and%20reporting%20of%20COVID%20PCR%20results.pdf.

[25] RAG Meeting 05/31/2021: Interpretation of RT-PCR Results of SARS-CoV-2 Traces. https://covid-19.sciensano.be/sites/default/files/Covid19/20210531_Advice_RAG_Interpretation%20of%20traces%20of%20SARS-CoV-2_NL.pdf.

[26] Laboratory Test Result Healthdata.be Sciensano. https://covid19lab.healthdata.be/data-collection/laboratorytestresult.

[27] Meurisse M., Lajot A., Dupont Y., Lesenfants M., Klamer S., Rebolledo J., Lernout T., Leroy M., Capron A., Van Bussel J. (2021). One year of laboratory-based COVID-19 surveillance system in Belgium: Main indicators and performance of the laboratories (March 2020–2021). Arch. Public Health.

[28] Planas D., Saunders N., Maes P., Guivel-Benhassine F., Planchais C., Buchrieser J., Bolland W.H., Porrot F., Staropoli I., Lemoine F. (2021). Considerable escape of SARS-CoV-2 Omicron to antibody neutralization. Nature.

[29] Bruel T., Hadjadj J., Maes P., Planas D., Seve A., Staropoli I., Guivel-Benhassine F., Porrot F., Bolland W.-H., Nguyen Y. (2022). Serum neutralization of SARS-CoV-2 Omicron sublineages BA.1 and BA.2 in patients receiving monoclonal antibodies. Nat. Med..

[30] Wawina-Bokalanga T., Martí-Carreras J., Vanmechelen B., Bloemen M., Wollants E., Laenen L., Cuypers L., Beuselinck K., Lagrou K., André E. (2021). Genetic diversity and evolution of SARS-CoV-2 in Belgium during the first wave outbreak. bioRxiv.

[31] Vanmechelen B., Logist A.-S., Wawina-Bokalanga T., Verlinden J., Martí-Carreras J., Geenen C., Slechten B., Cuypers L., André E., Baele G. (2022). Identification of the First SARS-CoV-2 Lineage B.1.1.529 Virus Detected in Europe. Microbiol. Resour. Announc..

[32] Janssen R., Laenen L., Cuypers L., Capron A., Beuselinck K., Van Ranst M., Lagrou K., André E., Dequeker E., COVID-19 Genomics Belgium Consortium (2022). Achieving quality assurance of high-throughput diagnostic molecular testing during the SARS-CoV-2 pandemic—role of the Belgian national reference laboratory for SARS-CoV-2.

[33] Van Vooren S., Grayson J., Van Ranst M., Dequeker E., Laenen L., Janssen R., Gillet L., Bureau F., Coppieters W., Devos N. (2022). Reliable and Scalable SARS-CoV-2 qPCR Testing at a High Sample Throughput: Lessons Learned from the Belgian Initiative. Life.

[34] Aksamentov I., Roemer C., Hodcroft E.B., Neher R.A. (2021). Nextclade: Clade assignment, mutation calling and quality control for viral genomes. J. Open Source Softw..

[35] O’Toole Á., Scher E., Underwood A., Jackson B., Hill V., McCrone J.T., Colquhoun R., Ruis C., Abu-Dahab K., Taylor B. (2021). Assignment of Epidemiological Lineages in an Emerging Pandemic Using the Pangolin Tool. Virus Evol..

[36] Dahdouh E., Lázaro-Perona F., Romero-Gómez M.P., Mingorance J., García-Rodriguez J. (2021). Ct values from SARS-CoV-2 diagnostic PCR assays should not be used as direct estimates of viral load. J. Infect..

[37] Rhoads D., Peaper D.R., She R.C., Nolte F.S., Wojewoda C.M., Anderson N.W., Pritt B.S. (2021). College of American Pathologists (CAP) Microbiology Committee Perspective: Caution Must Be Used in Interpreting the Cycle Threshold (Ct) Value. Clin. Infect. Dis..

[38] van Kasteren P.B., van Der Veer B., van den Brink S., Wijsman L., de Jonge J., van den Brandt A., Molenkamp R., Reusken C.B.E.M., Meijer A. (2020). Comparison of seven commercial RT-PCR diagnostic kits for COVID-19. J. Clin. Virol..

[39] Kanwar N., Banerjee D., Sasidharan A., Abdulhamid A., Larson M., Lee B., Selvarangan R., Liesman R.M. (2021). Comparison of Diagnostic Performance of Five Molecular Assays for Detection of SARS-CoV-2. Diagn. Microbiol. Infect. Dis..

[40] Ehret R., Breuer S., Dhein J., Reinhardt B., Obermeier M. (2022). Clinical evaluation of the automated Abbott RealTime SARS-CoV-2, Alinity m SARS-CoV-2, and Alinity m Resp-4-Plex assays. J. Virol. Methods.

[41] Bentley E., Mee E.T., Routley S., Mate R., Fritzsche M., Hurley M., Le Duff Y., Anderson R., Hockley J., Rigsby P. (2020). Collaborative Study for the Establishment of a WHO International Standard for SARS-CoV-2 RNA. Expert Committee on Biological Standardization.

[42] AACC Recommendation for Reporting SARS-CoV-2 Cycle Threshold (Ct) Values. https://www.aacc.org/science-and-research/covid-19-resources/statements-on-covid-19-testing/aacc-recommendation-for-reporting-sars-cov-2-cycle-threshold-ct-values.

[43] Sethuraman N., Jeremiah S.S., Ryo A. (2020). Interpreting Diagnostic Tests for SARS-CoV-2. JAMA.

[44] Payne D., Newton D., Evans P., Osman H., Baretto R. (2020). Preanalytical issues affecting the diagnosis of COVID-19. J. Clin. Pathol..

[45] Rahbari R., Moradi N., Abdi M. (2021). rRT-PCR for SARS-CoV-2: Analytical considerations. Clin. Chim. Acta.

[46] Gand M., Vanneste K., Thomas I., Van Gucht S., Capron A., Herman P., Roosens N., De Keersmaecker S. (2021). Deepening of in Silico Evaluation of SARS-CoV-2 Detection RT-qPCR Assays in the Context of New Variants. Genes.

[47] Wollschläger P., Todt D., Gerlitz N., Pfaender S., Bollinger T., Sing A., Dangel A., Ackermann N., Korn K., Ensser A. (2021). SARS-CoV-2 N gene dropout and N gene Ct value shift as indicator for the presence of B.1.1.7 lineage in a commercial multiplex PCR assay. Clin. Microbiol. Infect..

[48] Brown K.A., Gubbay J., Hopkins J., Patel S., Buchan S.A., Daneman N., Goneau L.W. (2021). S-Gene Target Failure as a Marker of Variant B.1.1.7 among SARS-CoV-2 Isolates in the Greater Toronto Area, December 2020 to March 2021. JAMA.

[49] Clark C., Schrecker J., Hardison M., Taitel M.S. (2022). Validation of reduced S-gene target performance and failure for rapid surveillance of SARS-CoV-2 variants. medRxiv.

[50] Mendes R.A. (2021). We need increased targeted measures now to slow the spread of omicron. BMJ.

[51] UK Health Security Agency (2021). SARS-CoV-2 Variants of Concern and Variants Under Investigation in England, Technical Briefing 32. https://assets.publishing.service.gov.uk/government/uploads/system/uploads/attachment_data/file/1042688/RA_Technical_Briefing_32_DRAFT_17_December_2021_2021_12_17.pdf.

[52] Walsh K.A., Jordan K., Clyne B., Rohde D., Drummond L., Byrne P., Ahern S., Carty P.G., O’Brien K.K., O’Murchu E. (2020). SARS-CoV-2 detection, viral load and infectivity over the course of an infection. J. Infect..

[53] Liu W.-D., Chang S.-Y., Wang J.-T., Tsai M.-J., Hung C.-C., Hsu C.-L., Chang S.-C. (2020). Prolonged virus shedding even after seroconversion in a patient with COVID-19. J. Infect..

[54] Quicke K., Gallichotte E., Sexton N., Young M., Janich A., Gahm G., Carlton E.J., Ehrhart N., Ebel G.D. (2020). Longitudinal surveillance for SARS-CoV-2 RNA among asymptomatic staff in five Colorado skilled nursing home facilities: Epidemiologic, virologic and sequence analysis. medRxiv.

[55] Baang J.H., Smith C., Mirabelli C., Valesano A.L., Manthei D.M., Bachman M.A., Wobus C.E., Adams M., Washer L., Martin E.T. (2020). Prolonged Severe Acute Respiratory Syndrome Coronavirus 2 Replication in an Immunocompromised Patient. J. Infect. Dis..

